# Mapping the Metabolic Niche of Citrate Metabolism and *SLC13A5*

**DOI:** 10.3390/metabo13030331

**Published:** 2023-02-23

**Authors:** Fangfang Chen, Hanna Friederike Willenbockel, Thekla Cordes

**Affiliations:** 1 Research Group Cellular Metabolism in Infection, Helmholtz Centre for Infection Research (HZI), 38124 Braunschweig, Germany; 2 Department of Bioinformatics and Biochemistry, Braunschweig Integrated Centre of Systems Biology (BRICS), Technische Universität Braunschweig, 38106 Braunschweig, Germany

**Keywords:** SLC13A5, NaCT, citrate metabolism, metabolic niche, tracing, mass spectrometry, TCA cycle, citrate transport, mitochondria, compartmentalization

## Abstract

The small molecule citrate is a key molecule that is synthesized de novo and involved in diverse biochemical pathways influencing cell metabolism and function. Citrate is highly abundant in the circulation, and cells take up extracellular citrate via the sodium-dependent plasma membrane transporter NaCT encoded by the *SLC13A5* gene. Citrate is critical to maintaining metabolic homeostasis and impaired NaCT activity is implicated in metabolic disorders. Though citrate is one of the best known and most studied metabolites in humans, little is known about the consequences of altered citrate uptake and metabolism. Here, we review recent findings on *SLC13A5*, NaCT, and citrate metabolism and discuss the effects on metabolic homeostasis and *SLC13A5*-dependent phenotypes. We discuss the “multiple-hit theory” and how stress factors induce metabolic reprogramming that may synergize with impaired NaCT activity to alter cell fate and function. Furthermore, we underline how citrate metabolism and compartmentalization can be quantified by combining mass spectrometry and tracing approaches. We also discuss species-specific differences and potential therapeutic implications of *SLC13A5* and NaCT. Understanding the synergistic impact of multiple stress factors on citrate metabolism may help to decipher the disease mechanisms associated with SLC13A5 citrate transport disorders.

## 1. Introduction

Citrate is present in almost all cell types and bodily fluids and is a critical energy sensor to maintain cellular metabolic homeostasis. It is involved in numerous metabolic pathways, including the tricarboxylic acid (TCA) cycle, glucose, and lipid metabolism as well as post-translational modification and metal chelating [1,2,3]. Alteration in citrate levels affects numerous metabolic pathways further influencing intracellular signaling in response to genetic and environmental changes such as immune responses [4,5]. Almost all cell types synthesize citrate de novo within the TCA cycle catalyzed by mitochondrial citrate synthase (CS) or in the cytosol via reductive carboxylation [4,5,6]. Mitochondrial citrate is also transported into the cytosol via the mitochondrial citrate transport protein (CTP). Alternatively, cells take up citrate from the environment via the sodium-coupled citrate transporter NaCT encoded by the gene *SLC13A5*. Of note, *SLC13A5* expression is cell-type specific and influenced by certain metabolic stress factors. Mutations in the *SLC13A5* gene are implicated in epilepsy and are associated with SLC13A5 citrate transporter disorder [7]. 

Though citrate is critical to maintaining metabolic homeostasis malfunction of NaCT activity and impaired citrate uptake oftentimes do not manifest in phenotypic changes indicating the involvement of metabolic compensation strategies. Genetic alterations may synergize with certain stress conditions, including metabolic, environmental, and individual factors. Thus, here we consider the concept of the “multiple-hit theory” that might be involved in disease pathogenesis. At the onset of disease, the “first hit” may be metabolic changes caused by genetic mutations, followed by the “second hit” including inflammatory factors, mitochondrial dysfunction, oxidative stress, and other factors that ultimately lead to the disease phenotype. Since the interaction of genetic and environmental factors influences the metabolic crosstalk between cells and tissues, the “multiple-hit theory” is now widely accepted in diverse disease models [8]. 

In this review, we highlight recent discoveries of the plasma membrane carrier NaCT focusing on citrate metabolism and SLC13A5 transporter disorder. We will underline the critical role of citrate in the complex metabolic network and discuss metabolic stress conditions influencing citrate metabolism. Finally, we will discuss species-specific differences and potential therapeutic implications. Our review emphasizes the impact of metabolic stress conditions that may synergize with genetic alterations leading to pathophysiological phenotypes associated with impaired NaCT activity metabolism and citrate metabolism.

## 2. Citrate Plasma Membrane Transporter NaCT

Mammalian cells are highly compartmentalized and the transport of small molecules across membranes is not well understood. While some molecules diffuse through membranes, the transport of most organic acids, including citrate, is facilitated by solute carriers (SLCs). Over 65 gene families encode over 400 human SLCs influencing a wide range of physiological and pharmacological processes [9]. Members of the plasma membrane SLC13 and mitochondrial SLC25 gene carrier family are linked to mitochondrial and neurological disorders [7,10,11,12,13]. The expression of *SLC13A5* is cell-type-specific and influenced by environmental factors, such as inflammation and oxidative stress [14,15]. *SLC13A5* is expressed primarily in the liver, testes, and salivary glands, as well as in the brain, bones, and teeth [2,16,17]. However, *SLC13A5* might be expressed in other cell types under specific stress conditions that have not been identified yet. 

In humans, the concentration of citrate in plasma is around 200 µM [2], and in the brain, it is around 400 µm [18]. Such high levels allow citrate to perform a variety of functions in the brain, such as acting as an energy product and regulating neuronal excitability via ion chelation. In the brain, astrocytes synthesize and release citrate which influences the metabolism of other brain cells [19]. Notably, 80% of the body’s citrate is stored in the bones to maintain hardness, and bone citrate concentration (around 20–80 μmol/g) is 50 times higher compared to most soft tissues [18]. Furthermore, *SLC13A5* expression supports the differentiation of human mesenchymal stem cells into osteoblasts [20] and is upregulated during mouse tooth development [21]. A recent study identified the involvement of citrate in mineral hydroxyapatite, linking *Slc13a5* to defects in the mineralization of teeth and bones in mice [22]. Therefore, *SLC13A5*, NaCT, and citrate metabolism are critical factors for bone and tooth development as well as brain metabolism. 

Citrate is highly abundant in plasma, and the activities of the bones, the kidneys, and the liver influence circulating citrate concentrations [23]. Notably, citrate is absent in many cell culture media [15] and the lack of environmental citrate in cultured cell models may influence metabolic homeostasis. Thus, observations from in vitro experiments may not be suitable to identify disease mechanisms associated with impaired NaCT activity and citrate uptake.

### 2.1. SLC13A5 Citrate Transporter Disorder in Patients

*SLC13A5* is a single-gene cause of a severe metabolic disorder that has received increasing attention in recent years. So far, there are five members of the SLC13 family, three of which are associated with transport functions linked to membrane depolarization and transport of di- and tricarboxylic intermediates of the TCA cycle in a Na^+^-coupled manner [2]. In the nervous system, SLC13A5 citrate transport disorders are associated with pediatric epilepsy, Kohlschütter–Tönz syndrome, and other brain disorders, leading to imbalanced metabolic homeostasis affecting brain citrate and acetyl-CoA metabolism as well as ionic homeostasis [7,12,13,24,25]. To date, over 40 naturally occurring mutations in the *SLC13A5* gene have been identified in around 100 patients [7,24]. All of these mutations lead to loss of function in NaCT activity with impaired uptake activity of extracellular citrate. Most patients present with severe motor and cognitive impairment, as well as bone mineralization and tooth enamelization [7,13]. 

Brown and colleagues characterized the non-neurologic health of patients between the age of 1–17 years demonstrating that SLC13A5 disorder patients grow normally during the first three years of life and have minor health complications outside of the nervous system [26]. The patients’ records indicated the presence of gastrointestinal problems and a wide variety of respiratory complaints, with single or no abnormal diagnoses in other organ systems including the liver, kidneys, and heart [26]. The few available data on adolescent patients depict a tendency to grow slowly; therefore, more data are needed to elucidate the impact on growth and non-neurological disorders in adult patients [26]. Elevated plasma citrate level is a marker for impaired NaCT activity but it may not correlate with the disease outcome [27,28]. Therefore, loss of citrate uptake may synergize with other stress factors and may lead to metabolic compensation in certain patients.

### 2.2. The Species-Specific Differences in the Functional Features of the NaCT Transporter

**Figure 1:**
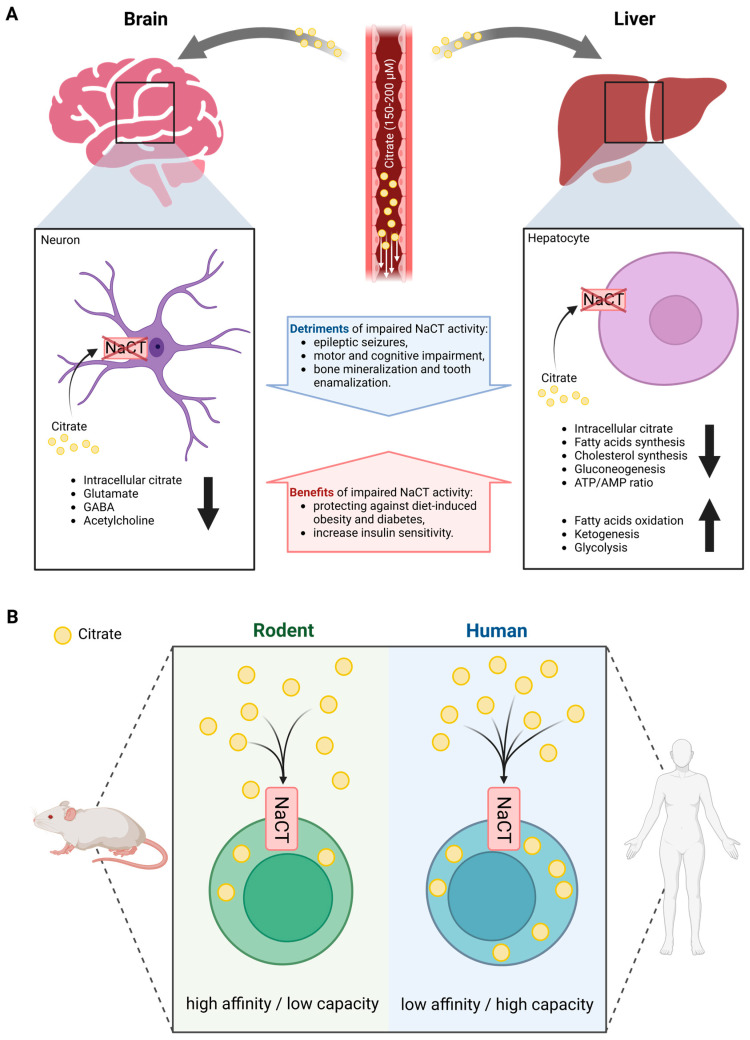
Overview of systemic citrate metabolism. (**A**) Organ-specific differences in phenotype outcomes of altered NaCT activity in mammals. (**B**) Species-specific differences in functional features of NaCT transport. (Created with BioRender.com).

Rogina et al. identified that a certain mutation in *Drosophila* led to a significantly extended lifespan [29]. Subsequently, they named this life-extending gene *Indy* and the protein INDY (I’m not dead yet). *Drosophila* INDY turned out to be a transporter with a preference for citrate over dicarboxylates and is the mammalian ortholog of NaCT [30]. Loss of function of NaCT activity leads to severe neurological dysfunction in humans, but to a less severe phenotype in mice, thus suggesting species-specific differences (Figure 1A) [31,32]. Indeed, human *SLC13A5* expression is highest in the liver followed by the brain and testes. In contrast, murine *Slc13a5* expression is more abundant in the brain and testes compared to the liver.

Patients with impaired NaCT activity have significantly elevated citrate levels in both plasma and cerebrospinal fluid (CSF), while *Slc13a5*-deficient mice have increased CSF citrate accumulation [28,33]. Citrate plasma levels were increased by two to three folds in patients with NaCT deficiency while levels in *Slc13a5*-deficient mice were highly variable. Contrary to humans, the deletion of *Slc13a5* in mice improved memory performance and motor coordination exhibiting increased neurogenesis [34]. Furthermore, mice with neuron-specific overexpression of *Slc13a5* exhibited autistic-like behaviors and impaired white matter integrity as well as modified synaptic structures [35]. Thus, species-specific differences in the functional features of the NaCT transporter may explain the differences observed between humans and mice (Figure 1B). In rodents, NaCT is a high-affinity/low-capacity transporter of citrate (*K*_M_ at 20–40 µM) [16,36], whereas human NaCT is a low-affinity/high-capacity transporter of citrate (*K*_M_ at 650–5000 µM) [14,16,37]. Based on the physiologic concentration of citrate in the circulation of 150–200 µM, rodent NaCT activity is expected to be close to saturation limiting citrate uptake capacity while human NaCT might not be saturated. Furthermore, the transport functions of human NaCT, in the presence of Na^+^, are activated markedly by Li^+^ while NaCT from non-primates is inhibited by Li^+^ [38,39]. 

Therefore, human NaCT may exhibit some distinctly different biochemical features and compensation strategies that may explain the different metabolic consequences of NaCT deficiency in different species. These findings raise suspicions about the utility of mouse models in assessing the biological functions of NaCT in patients. Hence, studies performed with mice may not reflect the physiological conditions occurring in humans.

## 3. Citrate Metabolism and NaCT 

**Figure 2:**
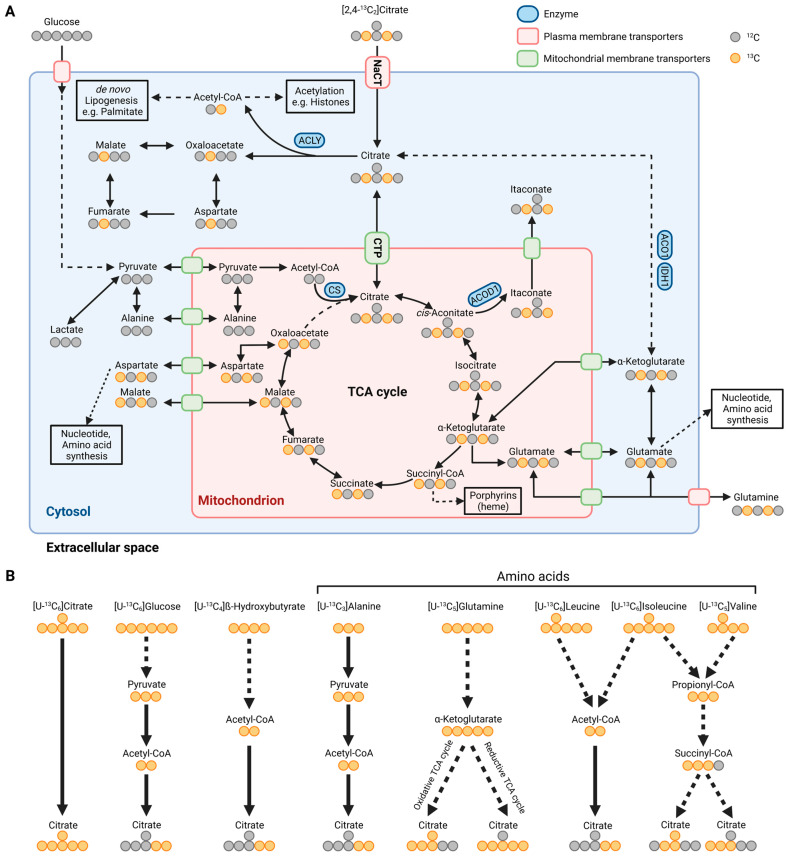
Metabolic map depicting carbon atom transitions and substrates fueling citrate metabolism. (**A**) Schematic of extracellular [2,4-^13^C_2_]citrate depicting carbon incorporation into tricarboxylic acid (TCA) cycle, lipogenesis, and amino acid synthesis in the cytosol and mitochondria. (**B**) Schematic depicting substrate utilization for citrate metabolism using [U-^13^C_6_]citrate, [U-^13^C_6_]glucose, [U-^13^C_4_]β-hydroxybutyrate, [U-^13^C_3_]alanine, [U-^13^C_5_]glutamine, [U-^13^C_6_]leucine, [U-^13^C_6_]isoleucine, and [U-^13^C_5_]valine. Labeled carbons are depicted in orange and unlabeled ones are depicted in grey. ACLY: ATP-citrate lyase; CS: citrate synthase; ACOD1: *cis*-aconitate decarboxylase; ACO1: aconitase 1; IDH1: isocitrate dehydrogenase 1; NaCT: sodium-coupled citrate transporter; CTP: mitochondrial citrate transport protein. (Created with BioRender.com).

**Figure 3:**
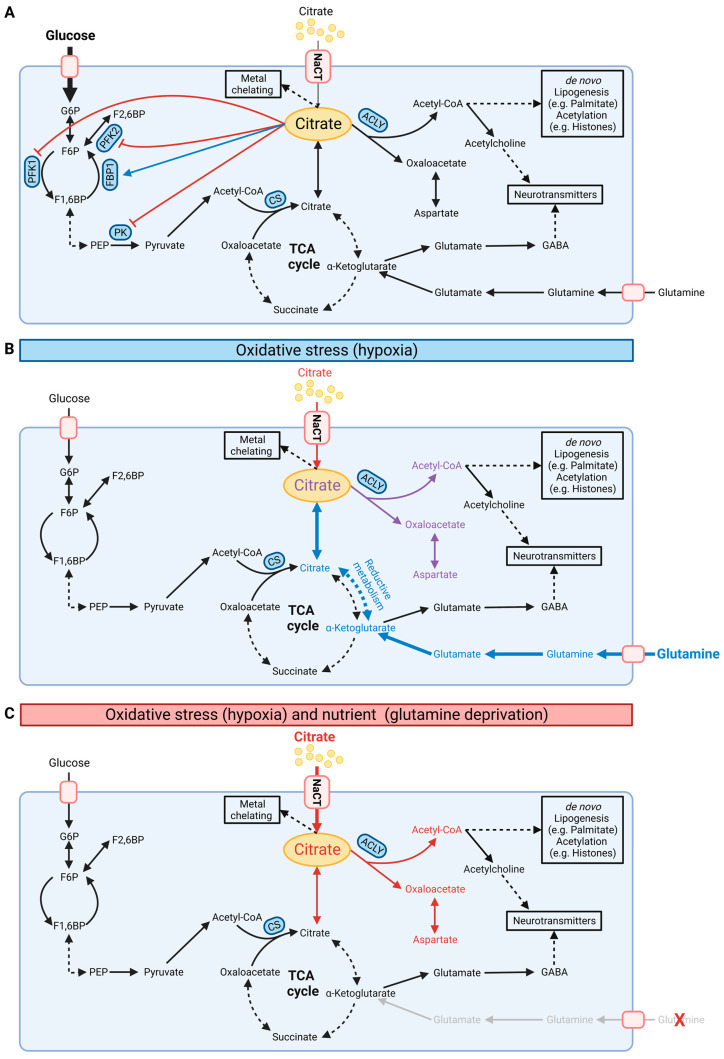
Schematic illustration of cellular citrate homeostasis. (**A**) Citrate is involved in multiple biochemical pathways under normoxic conditions. Cytosolic citrate inhibits glycolysis via PFK1, PFK2, and PK while stimulating FBP1 in gluconeogenesis. Cytosolic citrate is cleaved by ACLY to oxaloacetate and acetyl-CoA, thus influencing de novo lipogenesis, protein acetylation, and synthesis of neurotransmitters. (**B**) Under oxidative stress such as hypoxia conditions, cells reprogram citrate metabolism and increase reductive carboxylation metabolism for de novo lipogenesis. (**C**) NaCT facilitates citrate transport and metabolism under glutamine deprivation and oxidative stress. G6P: glucose-6-phosphate; F6P: fructose-6-phosphate; F1,6BP: fructose-1,6-bisphosphate; PEP: phosphoenolpyruvate; PFK: phosphofructokinase; PK: pyruvate kinase; FBP1: fructose 1,6-bisphosphatase; ACLY: ATP-citrate lyase; CS: citrate synthase; NaCT: sodium-coupled citrate transporter. Black, red, and blue fonts distinguish the carbon sources glucose, citrate, and glutamine, respectively. In (**B**), purple font indicates the combined carbon sources citrate and glutamine. Bold fonts and bold arrows indicate increasing fluxes. In (**A**), the blue arrow indicates stimulation and the red arrows inhibition. (Created with BioRender.com).

Mitochondrial citrate and other TCA cycle intermediates have emerged as master regulators of cellular homeostasis influencing diverse pathophysiological processes (Figure 2A and Figure 3A). The intracellular citrate pool is influenced by exogenous citrate uptake and secretion, as well as endogenous citrate synthesis and further breakdown. Thus, regulating the intracellular citrate pool is critical to maintaining metabolic homeostasis. 

### 3.1. Citrate Modulates Diverse Metabolic Pathways and Networks

Within the TCA cycle, CS catalyzes the synthesis of citrate from acetyl-CoA and oxaloacetate. Alteration in substrate utilization fueling the mitochondrial acetyl-CoA pool, including glucose, ketone bodies, and amino acids, subsequently affects citrate synthesis and associated mitochondrial processes, including redox metabolism, respiration, and amino acid synthesis [1]. Citrate is also a precursor for *cis*-aconitate that is decarboxylated to itaconate by *cis*-aconitate decarboxylase (ACOD1) during immune responses [40]. Itaconate plays a critical role in metabolic and functional reprogramming in inflammatory diseases, with antimicrobial, antiviral, anti-inflammatory, metabolic, and other immunomodulatory effects [41,42,43,44,45,46]. CTP facilitates citrate transport to the cytosol and impaired CTP activity influences itaconate levels and histone acetylation [47]. CTP has been identified in vitro as a mitochondrial transporter of itaconate [48] further highlighting the impact of citrate on immune metabolism.

Cytosolic citrate is a metabolic regulator that allosterically inhibits glycolysis by phosphofructokinase 1 (PFK1), phosphofructokinase 2 (PFK2), and pyruvate kinase (PK) while stimulating fructose 1,6-bisphosphatase (FBP1), a key regulatory enzyme in gluconeogenesis (Figure 3A) [49]. This feedback inhibition tightly links TCA cycle metabolism to glycolysis. Cytosolic citrate is cleaved by ATP-citrate lyase (ACLY) to acetyl-CoA and oxaloacetate. Acetyl-CoA is a building block for de novo lipogenesis (DNL) and other downstream processes, including protein acetylation (Figure 2A) [1,50,51]. Acetyl-CoA is not directly transported to the cytosol; thus, the citrate–malate shuttle provides a major source of cytosolic acetyl-CoA pools [52,53,54]. Further inhibition of hepatocyte ACLY reduces lipogenesis and dyslipidemia, improving non-alcoholic steatohepatitis (NASH) and liver fibrosis [55]. Of note, endoplasmic reticulum stress influences the expression of CTP suggesting the involvement of citrate in proteostasis [56]. Therefore, altering the citrate pool by NaCT and CTP activity affects acetyl-CoA pools and downstream metabolic pathways. 

Under certain stress conditions, citrate pools can also be fueled by alternative substrates. In oxidative stress situations, including impaired mitochondrial respiration or low oxygen availability, cells undergo drastic metabolic reprogramming and utilize glutamine for citrate synthesis via reductive carboxylation [6]. This reaction bypasses the decarboxylation step of α-ketoglutarate during oxidative TCA cycle metabolism and fuels the cytosolic citrate pool for DNL under hypoxic conditions. Hence, extracellular citrate uptake facilitated by NaCT may influence substrate utilization and metabolic reprogramming in response to stress factors. 

Furthermore, citrate is involved in neurotransmitter synthesis, including glutamate, γ-aminobutyric acid (GABA), and acetylcholine. Citrate contributes to cytosolic acetyl-CoA pools used for the synthesis of acetylcholine which is implicated in memory and cognitive function and motor control. GABA and glutamate are critical for memory and cognition [2]. Thus, impaired NaCT activity may affect levels of neurotransmitters modulating the pathogenesis of brain disorders. 

Collectively, citrate modulates critical biochemical reactions to maintain metabolic homeostasis. Key enzymes involved in the synthesis (isocitrate dehydrogenase (IDH) and CS), transport (NaCT and CTP), or breakdown (ACLY and IDH) of citrate are potential therapeutic targets as they influence disease pathogenesis, including brain disorders and cancer. However, more research is needed to identify genetic, environmental, and other stress factors influencing citrate metabolism. 

### 3.2. Citrate Metabolism under Oxidative Stress Conditions

Loss of function of NaCT activity causes severe epilepsy in humans while a less intense phenotype occurs in mice [7,25,36]. *Slc13a5*-deficient mice had an increased propensity for epileptic seizures and increased excitability in hippocampal neurons upon administration of pro-epileptic agents [33] suggesting that NaCT activity may be influenced by specific stress factors. Citrate contributes carbons to diverse metabolic pathways. Hence, reprogramming of substrate utilization under stress conditions may influence citrate uptake and utilization. Metabolism is highly reprogrammed under hypoxic conditions to compensate for reduced oxidative pathways (Figure 3). For instance, under hypoxic conditions, cells reprogram central carbon metabolism and increase glutamine utilization for the TCA cycle and DNL via reductive carboxylation [6]. Since most media lack citrate, in vitro metabolic studies are commonly performed with sub-physiological citrate levels. Recently, Kumar and colleagues demonstrated that exogenous citrate becomes a significant fuel for TCA cycle metabolism and lipogenesis only under hypoxic conditions in hepatocellular carcinoma (HCC) cells [15]. In primary brain cultures, citrate contributed to lipid metabolism, but less to TCA cycle metabolism in hypoxic conditions indicating cell-type-specific utilization of extracellular citrate. NaCT supports the input of extracellular citrate, which is mainly catabolized in the cytosol to produce acetyl-CoA for DNL influencing reductive carboxylation pathways. The critical citrate uptake under hypoxic conditions rescued HCC cell growth in glutamine-deprived conditions by fueling the TCA cycle and lipid metabolism [15]. Thus, citrate influences metabolic reprogramming under oxidative stress conditions that might be a metabolic vulnerability to limit cancer cell growth. 

In addition, citrate is a metal chelating agent and citrate uptake via NaCT potentiated the survival of HCC cells under Zn^2+^ treatment [15]. This finding is consistent with previous studies demonstrating that citrate protects neurons from the cytotoxicity of Zn^2+^ [3]. The zinc chelation might be involved in the pathophysiology of brain citrate, as reduced zinc levels may result in an excitation–inhibition imbalance influencing seizure susceptibility [11]. Furthermore, zinc regulates mitochondrial aconitase activity and high levels of citrate directly impact mitochondrial metabolism as observed in osteoblasts and prostate cancer [57,58]. Thus, extracellular citrate uptake influences diverse metabolic networks and biological processes, specifically in situations with imbalanced nutrient, oxygen, and metal availability.

### 3.3. Impact of Immune Response on SLC13A5

Citrate influences diverse metabolic pathways involved in immune responses but the impact of inflammation on *SLC13A5* and citrate uptake is not well understood. Thus, cell types expressing *SLC13A5*, including hepatocytes, may respond to inflammatory stimuli. For instance, non-alcoholic fatty liver disease (NAFLD) is associated with insulin resistance, inflammation, and liver cirrhosis resulting from altered fatty acid oxidation and energy uptake. Indeed, the knockdown of hepatic *Slc13a5* improved hepatic insulin sensitivity, prevented hepatic neutral lipid and triglyceride accumulation, and protected diet-induced NAFLD. Of note, plasma interleukin 6 (IL-6) levels decreased by more than 35% in *Slc13a5*-knockdown mice compared to wild-type mice [59], indicating the involvement of NaCT in immune responses.

IL-6 is a pleiotropic cytokine that regulates the immune response as well as hematopoiesis, metabolism, and organ development [60]. A direct link between IL-6 and *SLC13A5* levels has been reported in human NAFLD patients by von Loeffelholz [14]. Liver *Slc13a5* levels were increased in mice on a high-fat diet and in non-human primates on a 2-year high-fat, high-sucrose diet. It has been proposed that IL-6 is a regulator of *SLC13A5* via signal transducer and activator of transcription 3 (STAT3), and induction of *SLC13A5* expression was abolished by IL-6 receptor blockage [14]. Thus, NaCT and citrate link metabolism to inflammation via IL-6, and targeting *SLC13A5* may have therapeutic potential for metabolic disorders, including NAFLD. 

## 4. Quantifying Citrate Metabolism Using Mass Spectrometry and Tracing Approaches

A robust and accurate quantification of citrate in diverse biological materials is critical for diagnostic and therapeutic purposes. A gas or liquid chromatograph (GC or LC) coupled to mass spectrometry (MS) is commonly used to quantify citrate concentrations in biological samples from SLC13A5 patients and model systems [28,33,61]. Metabolism is highly dynamic, and quantifying metabolic levels does not reflect complex metabolic processes occurring in response to genetic or environmental changes. Therefore, mass spectrometry combined with tracing approaches has emerged as a versatile tool to quantify and visualize metabolic fluxes in highly dynamic, biological systems [42,62,63]. Radioactive tracers, such as ^14^C citrate are used to quantify metabolic uptake. However, stable isotopically labeled tracers offer a reliable and safe alternative to decipher metabolic fluxes in biological systems [15,64].

Endogenous citrate synthesis is influenced by substrates fueling carbons into the TCA cycle metabolism, such as amino acids and precursors of acetyl-CoA. Isotopically labeled carbon tracers, such as ^13^C glutamine and glucose tracers (Figure 2B) are commonly applied to quantify citrate metabolism [6,22,65]. Labeled fatty acids, ketone bodies, and amino acids further supply carbons for citrate synthesis (Figure 2B) [43,66,67,68,69,70]. More specific tracers, such as ^13^C and ^2^Hlabeled citrate, are advantageous to trace the fate of citrate in a living organism [15,71]. For example, while red blood cells (RBC) contain no mitochondria, citrate tracer approaches indicated citrate uptake and utilization in RBCs [71]. Notably, RBCs take up extracellular citrate via the mitochondrial transporter protein CTP located on the plasma membrane [71,72]. Thus, citrate uptake might be facilitated by alternative strategies including transporters that have not been annotated yet. 

Furthermore, Kumar et al. observed that the addition of unlabeled citrate into culture media containing uniformly (U) [U-^13^C_5_]glutamine or [U-^13^C_6_]glucose diluted the central carbon metabolism in cultured HCC and neuronal cells. To visualize the fate of citrate in more detail, they applied [2,4-^13^C_2_]citrate and observed that citrate contributed labeled carbons to TCA cycle metabolism, with significantly higher enrichment under hypoxic conditions (Figure 2A). They also observed significant enrichment on palmitate indicating that citrate fuels carbons into the acetyl-CoA pool for DNL (Figure 3B,C). Of note, the positional labeling of substrates downstream of citrate, specifically labeling on aspartate, malate, and α-ketoglutarate (Figure 2A), provides additional information on compartmentalized citrate metabolism. The authors generated a ^13^C metabolic flux analysis model to address the spatial intracellular fluxes, further demonstrating that exogenous citrate is metabolized primarily in the cytosol via ACLY and aconitase 1 (ACO1)/IDH1 activity (Figure 3B,C). Genetically encoded biosensors to image cytosolic and mitochondrial citrate concentrations are now available and it would be useful to apply these biosensors in situations with impaired NaCT activity [73]. 

Thus, metabolic flux analysis and biosensor approaches may help to decipher the spatial impact of citrate metabolism in disease mechanisms associated with impaired NaCT activity. Diverse techniques, including mass spectrometry, are commonly used to quantify citrate levels in tissues, bodily fluids, and cultured cells. Though citrate is a biomarker in NaCT-related disorders, citrate is still not included in metabolic panels of standard blood tests.

## 5. Therapeutic Potential of *SLC13A5*/NaCT

Citrate uptake is tissue-type specific and we are just beginning to understand how NaCT is regulated under metabolic stress conditions. Recently, Li and Wang discussed the molecular mechanisms influencing *SLC13A5* expression, including the transcription factors STAT3 and cAMP-responsive element-binding protein (CREB) [74]. Expression of *SLC13A5* was elevated in patients with NAFLD, obesity, and type II diabetes [74,75]. Xenobiotic chemical challenges such as lipopolysaccharide (LPS), benzo[a]pyrene (BaP), or rifampicin (RIF) induced the expression of *SLC13A5* as well as the hormone glucagon and the cytokine IL-6 [14,76,77,78]. Furthermore, impaired NaCT function prevents high-fat diet-induced insulin resistance, alters lipid metabolism [36,79], and correlates with the outcomes of NAFLD patients [14]. In addition to these beneficial metabolic effects, NaCT inhibition in mice reduces peripheral catecholamine biosynthesis, which attenuates the sympathetic nervous system to improve blood pressure and heart rate control, thereby reducing arterial hypertension [80]. Thus, the development of chemical NaCT inhibitors has attracted increasing therapeutic interest [81]. 

The inhibitor PF-06649298 has negligible species selectivity for mice and humans with an IC_50_ of 0.4–16.2 µM [79,82]. PF-06649298 and its derivative PF-06761281 were allosteric, state-dependent inhibitors for NaCT; their inhibitory potency was higher with increasing citrate concentration [83]. BI01383298 is human species selective and irreversibly inhibits NaCT with an IC_50_ of 24–56 nM [31]. More recently, Zahn and colleagues introduced the molecule ETG-5773, a cross-species, non-competitive inhibitor of NaCT with an IC_50_ of 160 nM for humans and 180 nM for mice [84]. Administration of ETG-5773 to diet-induced obesity mice decreased body weight, blood glucose, insulin, and liver triglycerides levels and improved glucose tolerance [84]. The pharmacological outcome of the inhibitors reflects the metabolic changes observed in *Slc13a5*-deficient mice [14,36] and they might be promising candidates for modulating NaCT activity. Furthermore, adeno-associated virus (AAV)-based gene replacement therapies [85,86] may be a promising approach for the treatment of patients with impaired NaCT activity but more clinical data are critically needed. To date, effective therapies are severely limited and treatment strategies based on a ketogenic diet or valproate have different outcomes in patients with *SLC13A5* deficiency [27,87]. Thus, future treatment strategies must also consider individualized medical approaches.

## 6. Conclusions

Alteration in NaCT carrier activity has emerged as a metabolic regulator influencing cell metabolism and function. We are only beginning to understand the impact of altered NaCT activity and citrate metabolism on cellular homeostasis. Since citrate plays a different role in the mitochondria and cytoplasm, it is important to decipher the spatial consequences of citrate metabolism. It remains unclear whether decreased intracellular or increased extracellular citrate levels are the driver in the pathogenesis of diseases associated with impaired NaCT activity. Since NaCT also facilitates the uptake of small molecules beyond citrate, NaCT may influence biochemical pathways independent of citrate uptake [16,88]. A limiting factor in advancing our understanding of SLC13A5 diseases is the lack of model systems reflecting physiological conditions occurring in patients. More insights into clinical data are needed to better understand the species-specific impact to improve therapeutic implications. Factors contributing to different outcomes of clinical therapies are not well understood, thus further supporting consideration of the “multiple-hit theory”. Most SLC13A5 patients develop seizures within the first day of life, possibly due to the dramatic change in the environment after birth. At the onset of the disease, the “first hit” is *SLC13A5* deficiency, with them being unable to transport extracellular citrate into their cells, followed by multiple other hits of various stresses that eventually lead to the SLC13A5 disease phenotype. Thus, while genetic or environmental factors alone may not influence cell function, a combination of them may synergize with other factors leading to pathophysiological phenotypes. Therefore, understanding the synergistic impact of combined metabolic stresses may help to develop treatment strategies for patients with impaired citrate metabolism.

## Abbreviations

^12^C	Carbon-12
^13^C	Carbon-13
^14^C	Carbon-14
[U-^13^C]substrate	Uniformly labeled ^13^C carbon tracer
AAV	Adeno-associated virus
ACLY	ATP-citrate lyase
ACO1	Aconitase 1
ACOD1	*cis*-Aconitate decarboxylase
ATP	Adenosine triphosphate
BaP	Benzo[a]pyrene
CoA	Coenzyme A
CREB	cAMP-responsive element-binding protein
CS	Citrate synthase
CSF	Cerebrospinal fluid
CTP	Mitochondrial citrate transport protein
DNL	De novo lipogenesis
F1,6BP	Fructose-1,6-bisphosphate
F6P	Fructose-6-phosphate
FBP1	Fructose 1,6-bisphosphatase
G6P	Glucose-6-phosphate
GABA	γ-aminobutyric acid
GC	Gas chromatograph
HCC	Hepatocellular carcinoma
HIF-1α	Hypoxia-inducible factor 1-alpha
IC_50_	The half-maximal inhibitory concentration
IDH	Isocitrate dehydrogenase
IL-6	Interleukin 6
INDY	I’m Not Dead Yet
*K*_M_	Michaelis constant
LC	Liquid chromatograph
Li^+^	Lithium ion
LPS	Lipopolysaccharide
MS	Mass spectrometry
Na^+^	Sodium ion
NaCT	Sodium-coupled citrate transporter (protein)
NAFLD	Non-alcoholic fatty liver disease
NASH	Non-alcoholic steatohepatitis
PEP	Phosphoenolpyruvate
PFK1	Phosphofructokinase 1
PFK2	Phosphofructokinase 2
PK	Pyruvate kinase
RBC	Red blood cell
RIF	Rifampicin
SLCs	Solute carriers
SLC13A5	Solute carrier family 13 member 5
*SLC13A5*	Solute carrier family 13 member 5 (human gene)
*Slc13a5*	Solute carrier family 13 member 5 (mouse gene)
SLC25A1	Solute carrier family 25 member 1
STAT3	Signal transducer and activator of transcription 3
TCA	Tricarboxylic acid
Zn^2+^	Zinc ions
